# Problematic smartphone use and affective temperaments among Lebanese young adults: scale validation and mediating role of self-esteem

**DOI:** 10.1186/s40359-021-00638-y

**Published:** 2021-09-08

**Authors:** Joanne Zeidan, Souheil Hallit, Marwan Akel, Ismail Louragli, Sahar Obeid

**Affiliations:** 1 Faculty of Arts and Sciences, Holy Spirit University of Kaslik (USEK), Jounieh, Lebanon; 2 Faculty of Medicine and Medical Sciences, Holy Spirit University of Kaslik (USEK), Jounieh, Lebanon; 3 INSPECT-LB: National Institute of Public Health, Clinical Epidemiology and Toxicology, Beirut, Lebanon; 4 School of Pharmacy, Lebanese International University, Beirut, Lebanon; 5 Ibn Tofail University, Kenitra, Morocco; 6 Research Department, Psychiatric Hospital of the Cross, Jal Eddib, Lebanon

**Keywords:** Problematic use of smartphones, Self-esteem, Affective temperaments, Lebanese adults, Smartphone addiction

## Abstract

**Background:**

Adults all over the world face serious issues from problematic smartphone use (PSU). It influences them negatively on a cognitive, behavioral, and emotional level, as well as on their tendencies and well-being. In Lebanon, the prevalence of PSU was shown to be 20.2% within the adult population, specifically with young adults (18–34 years old). This study investigates the validity and reliability of the Smartphone Addiction Scale (SAS) Arabic version. In addition, this study evaluates the association between PSU and affective temperaments and the mediating role of self-esteem in this association.

**Method:**

A cross-sectional study was carried out between August and September 2020, using a sample of community-dwelling participants aged 18 to 29 years. The Smartphone Addiction Scale—Short Version was used to evaluate smartphone addiction among adolescents and adults. The five different temperaments of the patients were assessed by using the Affective temperament Scale (TEMPS‐A). The Rosenberg Self-Esteem Scale was used to evaluate self-esteem.

**Results:**

461 participants were included in this study. All items of the SAS were extracted and yielded a one-factor solution with Eigenvalues > 1 (variance explained = 49.96%; α_Cronbach_ = 0.886). The confirmatory analysis results consolidated those obtained from the factor analysis. Higher depressive temperament (B = 0.46) was significantly associated with more smartphone addiction, whereas higher self-esteem (B =  − 0.28) was significantly associated with less smartphone addiction. Self-esteem was found to mediate the association between depressive and hyperthymic temperaments with smartphone addiction.

**Conclusion:**

This study added a better understanding of the high smartphone addiction rate among adults in Lebanon. It confirms the association between affective temperaments and PSU through the mediating effect of self-esteem on Lebanese adults.

**Supplementary Information:**

The online version contains supplementary material available at 10.1186/s40359-021-00638-y.

## Background

A smartphone is “a device that combines a cell phone with a handheld computer, typically offering internet access, data storage, email capability, etc.” [1]. In the past few years, recent research explored the relationship that develops between the user and his smartphone [2–5]. Despite providing many advantages in improving the user’s everyday life, studies have shown that, in some situations, smartphones present association with patterns of behavioral addiction that lead to negative consequences [3, 8, 9]. Behavioral addiction is defined as “a repetitive habit pattern that increases the risk of disease and/or associated personal and social problems” [6]. In fact, previous studies have shown many examples of problematic use of smartphones leading to decrease of productivity, compromise of interactions with others, or mental illness [7–11], memory problems [12], alcohol use disorder [10, 13, 14], loneliness [15], alexithymia [16], etc.). To add, restriction from phone interaction in young adults can lead to performance impairment during a cognitive task and severe increases in levels of stress and anxiety [17–21].

Many studies use the term “smartphone addiction” based on the commonalities observed between excessive smartphone users and substance abusers such as losing control or seeking treatment [20, 21]. However, there is little to no evidence to this day that a problematic use of smartphone could in fact be considered as an addiction since factors like tolerance and withdrawal syndrome are yet to be explored in this matter [22]. To avoid overpathologizing, the term problematic smartphone use (PSU) is commonly used [23]. Billieux et al. developed a definition for PSU as “an inability to regulate one’s use of the smartphone, which eventually involves negative consequences in daily life” creating problems on social, psychological and behavioral levels [22–24]. The notion of PSU has its origins from two related notions. The first concept is that of the “problematic mobile phone use” introduced in recent research [25–27], especially before the revolutionary introduction of the smartphone in the market [9, 28, 29]. The second concept is the “problematic internet use” also known as “internet addiction” that helped in developing theories and methodologies for research work concerning PSU [30–32]. It is also worth mentioning that PSU is not acknowledged as a clinical form of addiction by the *Diagnostic and Statistical Manual for Mental Disorders (DSM-5)* and that it is, currently, still being debated about whether or not it should be recognized as such [33].

Adults all over the world face serious issues from PSU. It influences them negatively on a cognitive, behavioral, emotional level, and well-being [34]. PSU also constitutes a significant risk factor to people who are depressed or feeling lonely, anxious, and suffer from disturbance in their sleeping patterns [35]. To add, PSU may also cause problems in interpersonal relations and disturbances in academic performance [36–39].

The screening of PSU is performed using the short version of the Smartphone Addiction Scale (SAS-SV). The SAS is indeed a modern scale used to evaluate PSU validated amongst adolescents and young adults [40–45]. It was first developed in Korean language before being published in English [41]. The SAS-SV is one of the most validly used scales that is translated in different languages such as in Turkish [46], Spanish [47], French [47], Italian [43], Chinese [42] and Arabic [44, 48]. Despite its validated translation in Arabic in both Morocco [44] and Egypt [48], no psychometric validation of this scale has been carried out in Lebanon.

On that note, research found that people suffering from mood disorders are very prone to develop PSU [49]. Odegaard et al. [50] showed that in the early phases of major depressive disorders and bipolar disorders, early development of mood disorders is also affirmed by the presence of an affective temperament. This brings us to look into the association between PSU and affective temperaments. In fact, temperaments are unique characteristics of one’s personality, which do not generally change with time [51]. Many studies contributed into developing these five main affective temperaments: depressive, cyclothymic, hyperthymic, irritable and anxious [51, 52]. Previous research has shown that there is a relation between addiction to the Internet and all five affective temperaments, particularly with the anxious temperament [53].

While one of the factors involved in the development of PSU is affective temperaments, other studies link these two variables to self-esteem. One study highlighted the relationship between affective temperaments and self-esteem by showing the positive correlation between a high level of self-esteem and cyclothymic and anxious temperament [54]. Moreover, another research suggested sub-factors to each of the five affective temperaments and placed low self-esteem as a sub-factor to the depressive temperament while high self-esteem was placed as a sub-factor of the hyperthymic temperament [55]. Furthermore, other studies found that self-esteem is a risk factor and an antecedent of PSU [24, 56, 57].

In Lebanon, the prevalence of PSU was shown to be 20.2% with 95% CI [14.7, 25.7] within the adult population, specifically with young adults (18–34 years old) who are single and have access to mobile internet services [58]. In fact, since the fall of 2019, Lebanon saw the rise of an economic crisis as a consequence of poor political governance after the civil war. This caused different issues with the supply chains and decreased the Lebanese currency value vis-à-vis the US dollars. Lebanon suffered limitations on transferring money outside the country because of capital control done illegally [59]. Hence, unemployment rates grew with huge loss of purchasing capacity [60]. To add, the COVID-19 outbreak did not make things easier on the Lebanese population facing this hard economic crisis. A recent Lebanese study showed that fear created from the COVID-19 pandemic combined with the very difficult financial situation in the country was associated with high levels of stress and anxiety [61]. Another study done in Lebanon showed in fact that depression and anxiety are factors associated with PSU [62].

As we see, exploring PSU with an etiological intent can give us crucial results that could be used in order to diminish the negative consequences of PSU amongst Lebanese young adults. Even though research showed a relationship between affective temperaments and PSU, very little to no research backs up the underlying mechanism of this dynamic. Thus, the importance of clarifying the nature of this relationship lies in bringing a better understanding of the mediating effect that self-esteem has between affective temperaments and the development of PSU. While no previous studies have worked on the relationship between these three variables and of self-esteem as a mediating factor, this study investigates the reliability and validity of the Arabic version of the Smartphone Addiction Scale. Also, this study evaluates the association between PSU and affective temperaments, as well as the mediating effect of self-esteem.

## Methods

### Study design

A cross-sectional study was carried out between August and September 2020, during the lockdown period imposed by the government for the COVID-19 pandemic which coincides with the summer season vacation for most Lebanese. A sample of community-dwelling participants aged 18 to 29 years was used [63]. All methods were performed in accordance with the relevant guidelines and regulations.

### Participants

Due to the imposed COVID-19 restrictions, a survey was created using Google forms in order to mitigate the risky face-to-face interactions. The survey was shared among the participants and sent to all districts/governorates of Lebanon (Beirut, Mount Lebanon, North Lebanon, South Lebanon, and Bekaa) using the snowball technique; participants were asked to fill the survey online and share the link with other acquaintances and family members. All participants above 18 and below 30 years of age who had a mobile phone, were eligible to participate. Excluded were those who refused to fill out the questionnaire. Participants were not compensated for participation. A total of 461 persons participated in the study. Their mean age was 22.25 ± 2.87 years, with 70.9% females. Almost all the participants were single (91.3%) and have a university education level (94.4%). The mean household crowding index was 1.08 ± 0.61 (Table 1).

**Table 1:** Sociodemographic characteristics of the study sample (N = 461)

	Frequency (%)
*Gender*	
Male	134 (29.1%)
Female	327 (70.9%)
*Marital status*	
Single/widowed/divorced	421 (91.3%)
Married	40 (8.7%)
*Education level*	
School education	26 (5.6%)
University education	435 (94.4%)

	Mean ± SD
Age (in years)	22.25 ± 2.87
Household crowding index	1.08 ± 0.61

### Minimal sample size calculation

According to the G-power software, and based on an effect size f2 = 2%, an alpha error of 5%, a power of 80%, and taking into consideration 10 factors to be entered in the multivariable analysis, the results showed that a minimal number of 395 was needed.

### Translation procedure

The scales were forward and back-translated. Forward translation (English to Arabic) was performed by one translator, whereas the back translation from Arabic to English was performed by a second translator. Minor discrepancies were solved by consensus.

### Questionnaire and variables

The self-administered questionnaire with closed-ended questions was anonymous and available in Arabic and English; the questionnaire required approximately 25–30 min to be completed. The questionnaire consisted of different sections. The first part clarified socio-demographic characteristics: age, gender, marital status, work status, educational level, and household crowding index. The latter was calculated by dividing the number of persons in the house by the number of rooms in the house (excluding the bathrooms and kitchen); higher scores reflect lower socioeconomic status [64]. The second part of the questionnaire included the following scales:

#### Smartphone addiction scale-short version (SAS-SV)

The SAS-SV is a ten-item scale used to evaluate smartphone addiction among adolescents and adults [41, 42]. The total score was computed by adding the answers of these 10 items, with higher scores reflecting higher smartphone addiction. In this study, the SAS Cronbach’s alpha value was 0.886.

#### Affective temperament scale (TEMPS‐M)

Affective temperament traits were assessed by means of the brief version of the Temperament Evaluation of Memphis, Pisa, Paris, and San Diego (TEMPS-M) [65]. This scale is composed of 35 self-rating items that can be assigned to 5 subscales: depressive, cyclothymic, hyperthymic, irritable, and anxious. All responses are provided on 6-point Likert scales ranging from 1 (not at all) to 5 (very much). Each subscale score ranges from 5 to 35, with higher scores denoting higher expressions of the respective temperament. The Cronbach’s alpha values for each subscale were as follows: depressive (0.809), cyclothymic (0.898), hyperthymic (0.818), irritable (0.808), and anxious (0.856).

#### Rosenberg self‐esteem scale

The Rosenberg Self-Esteem Scale (RSES) is a 10-item scale that reflects self-worth by focusing on both positive and negative feelings people have about themselves [66]. Responses were scored from 1, meaning strongly disagree, to 4, meaning strongly agree, where higher scores reflect a better self-esteem (Cronbach’s alpha in this study 0.837).

### Statistical analysis

Data analysis was conducted using SPSS software v.23 and SPSS AMOS v.24 was used to conduct the confirmatory factor analysis. The latter was conducted based on the one-factor solution of the SAS items obtained in the original of the scale [41, 42]. The goodness-of-fit of the model was evaluated by calculating the root mean square error of approximation (RMSEA) statistic and the comparative fit index (CFI) as these are the most commonly used indices [67]. Values of RMSEA < 0.05 and < 0.11 indicate a close fit and an acceptable fit, respectively [68]. CFI values > 0.90 indicate a relatively good fit of the model [67].

Normal distribution of the SAS score was confirmed after calculating skewness and kurtosis; a range of value between − 2 and + 2 for asymmetry and kurtosis is adopted in order to prove normal univariate distribution [69]. These conditions consolidate the assumptions of normality in samples larger than 300 [70]. Student’s t-test was used to compare mean smartphone addiction differences between gender, marital status and education level respectively, whereas Pearson correlation coefficient was obtained to check for any correlation between two continuous variables (e.g. smartphone addiction and each temperament). A forward linear regression was conducted to check for correlates associated with smartphone addiction.

### Mediation analysis

The PROCESS SPSS Macro version 3.4, model four was used to calculate three pathways [71]. Pathway A determined the regression coefficient for the effect of each temperament on self-esteem. Pathway B examined the effect of self-esteem on smartphone addiction independent of temperament. Pathway C estimated the total and direct effect of each temperament on smartphone addiction and pathway AB calculated the indirect intervention effects. A 95% CI was used to assess for indirect effect significance [71]. Independent variables with a value of *p* < 0.2 in the bivariate analysis were included in the multivariable and mediation models. Cronbach’s alpha values were recorded for reliability analysis of all scales and subscales. Statistical significance was set at a *p* < 0.05.

## Results

The means and standard deviations of the scales were as follows: smartphone addiction (31.19 ± 8.80; median = 31), depressive temperament (16.13 ± 5.54), cyclothymic temperament (18.30 ± 6.94), hyperthymic temperament (20.69 ± 5.94), irritable temperament (18.38 ± 5.67), anxious temperament (17.32 ± 6.49), and self-esteem (28.48 ± 5.36).

### Confirmatory factor analysis

Table 2 presents the coefficients with standard errors and *p*-values of the direct effects of variables on each other. For this model, the estimated RMSEA is 0.09 with a confidence interval (0.082–0.109), which shows an acceptable fit, with a CFI value of 0.923, which shows adequate fit.

**Table 2:** Coefficient, standard error and *p*-value of the confirmatory factor analysis of the SAS scale items

Variable	Coefficient	Standard error	*p*
1. Missing planned work due to smartphone use	1		
2. Having a hard time concentrating in class, while doing assignments, or while working due to smartphone use	0.650	0.951	< 0.001
3. Feeling pain in the wrists or at the back of the neck while using a smartphone	0.478	0.741	< 0.001
4. Won’t be able to stand not having a smartphone	0.691	1.131	< 0.001
5. Feeling impatient and fretful when I am not holding my smartphone	0.707	1.043	< 0.001
6. Having my smartphone in my mind even when I am not using it	0.519	0.751	< 0.001
7. I will never give up using my smartphone even when my daily life is already greatly affected by it	0.689	1.072	< 0.001
8. Constantly checking my smartphone so as not to miss conversations between other people on Twitter or Facebook	0.696	0.994	< 0.001
9. Using my smartphone longer than I had intended	0.792	1.178	< 0.001
10. The people around me tell me that I use my smartphone too much	0.762	1.077	< 0.001

### Bivariate analysis

Higher depressive temperament (r = 0.358), cyclothymic temperament (r = 0.330), irritable temperament (r = 0.227), anxious temperament (r = 0.272) were significantly associated with more smartphone addiction, whereas higher self-esteem (r =  − 0.286) and higher household crowding index (r =  − 0.106) were significantly associated with less smartphone addiction (Table 3).

**Table 3:** Bivariate analysis taking the smartphone addiction scale as the dependent variable

	Smartphone addiction	*p*
	*Mean ± SD*	
*Gender*		
Male	31.06 ± 9.46	0.838
Female	31.24 ± 8.54	
*Marital status*		
Single	31.35 ± 8.82	0.198
Married	29.47 ± 8.58	
*Education level*		
School education	31.73 ± 9.29	0.748
University education	31.16 ± 8.79	
	*Correlation coefficient*	
Depressive temperament	0.358	** < 0.001**

Cyclothymic temperament	0.330	** < 0.001**
Hyperthymic temperament	0.091	0.051
Irritable temperament	0.227	** < 0.001**
Anxious temperament	0.272	** < 0.001**
Self-esteem scale	− 0.286	** < 0.001**
Age	− 0.052	0.266
Household crowding index	− 0.106	**0.023**
Number of children	0.012	0.804

Numbers in bold indicate significant *p*-values

### Multivariable analysis

The results of the linear regression, considering the smartphone addiction score as the dependent variable, showed that higher depressive temperament (B = 0.46) was significantly associated with more smartphone addiction, whereas higher self-esteem (B =  − 0.28) was significantly associated with less smartphone addiction (Table 4).

**Table 4:** Multivariable analysis: Linear regression considering the smartphone addiction score as the dependent variable

Variable	Unstandardized beta	Standardized beta	*p*	95% Confidence interval	
Depressive temperament	0.46	0.29	** < 0.001**	0.31	0.61
Self-esteem	− 0.28	− 0.17	** < 0.001**	-0.43	-0.13

Variables entered in the models: marital status, age, gender, household crowding index, depressive temperament, cyclothymic temperament, hyperthymic temperament, irritable temperament, anxious temperament and self-esteem scale

Adjusted R^2^ = 0.152, *p* < 0.001

Numbers in bold indicate significant *p*-values

### Mediation analysis

The direct and indirect effects of the associations between each temperament, self-esteem and smartphone addiction are summarized in Table 5. The detailed results of the mediation analysis are summarized in Additional file 1: Table 1.

**Table 5:** Mediation analysis: Direct and indirect effects of the associations between each temperament, self-esteem and smartphone addiction

Temperament	Direct effect			Indirect effect		
	Effect	SE	*p*	Effect	SE	95% BCa
Depressive	0.210	0.132	0.112	0.126	0.055	0.027–0.242
Cyclothymic	0.144	0.102	0.157	0.053	0.025	0.010–0.110
Hyperthymic	− 0.072	0.104	0.491	− 0.155	0.061	− 0.279 to − 0.039
Irritable	0.119	0.107	0.270	0.0004	0.019	− 0.042–0.038
Anxious	0.078	0.083	0.350	− 0.001	0.017	− 0.003–0.004

Direct effect = Effect of the temperament on smartphone addiction in the absence of the mediator (self-esteem); Indirect effect = Effect of the temperament on smartphone addiction in the presence of the mediator (self-esteem); SE = Standard Error; BCa = Bootstrap Confidence Interval

**Fig. 1:**
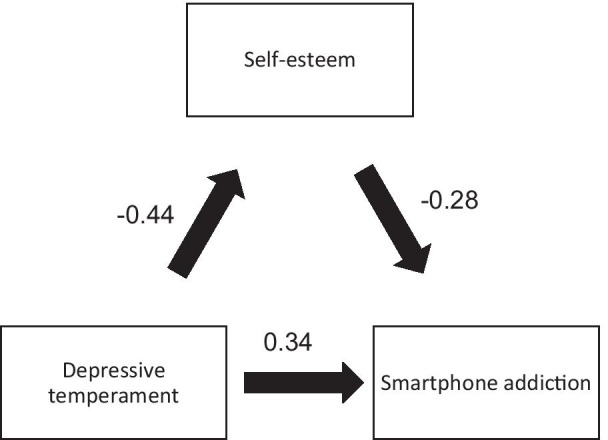
**a** Relation between depressive temperament and self-esteem; **b** Relation between self-esteem and smartphone addiction; **c’** Relation between depressive temperament and smartphone addiction

Depressive temperament was significantly associated with lower self-esteem and higher SAS score. When adding self-esteem to the model, depressive temperament did not show statistical significance with the SAS score, whereas higher self-esteem was significantly associated with lower SAS score (Fig. 1).

**Fig. 2:**
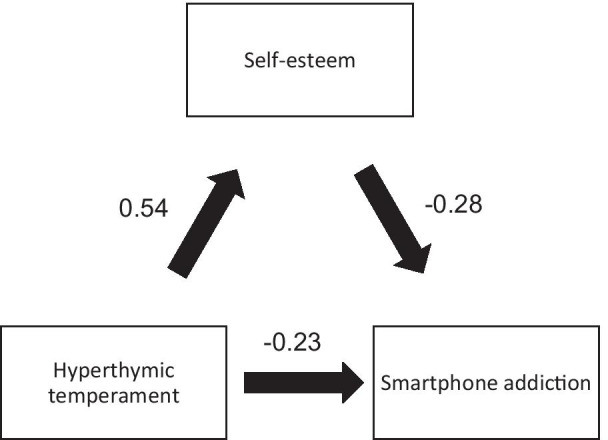
**a** Relation between hyperthymic temperament and self-esteem; **b** Relation between self-esteem and smartphone addiction; **c’** Relation between hyperthymic temperament and smartphone addiction

More hyperthymic temperament was significantly associated with higher self-esteem and lower SAS score. When adding self-esteem to the model, hyperthymic temperament did not show significance with the SAS score, whereas higher self-esteem was significantly associated with a lower SAS score (Fig. 2).

Self-esteem mediated the association between depressive temperament and smartphone addiction and between hyperthymic temperament and smartphone addiction.

## Discussion

### Validation of smartphone addiction scale short version (SAS-SV) in Arabic language

The confirmatory factor analysis results obtained in this study confirmed the one-factor solution of the SAS obtained in previous studies [41, 42]. The RMSEA value had an acceptable fit, whereas the CFI value suggested a good fitting model. The translated SAS-SV in this study was validated with a Cronbach's alpha coefficient of (α = 0.886). According to other studies that validated SAS-SV in their Arabic language populations, a study conducted by Sfendla et al., 2018 [44] on a sample of 310 university students in the Moroccan population showed an alpha coefficient of Cronbach of (α = 0.87), while another study by Elkholy et al., 2020 with 200 university students in Egyptian population obtained a Cronbach's alpha coefficient of (α = 0.94) [72]. However, this study’s reliability is higher than other studies such as those carried out by Andrade et al., 2020 in Brazilian version with (α = 0.819) [73] and Ching et al. 2020 in Malaise version with (α = 0.80) [40]. Thus, the Arabic version of the Smartphone Addiction Scale is similar to the original version and is a reliable tool for Lebanese young adults.

### Temperaments, self-esteem and PSU

The results of the linear regression, taking the smartphone addiction score as the dependent variable, showed that higher depressive temperament was significantly associated with more smartphone addiction. Ozturk et al. reported in their study that Internet addiction was highly associated with affective temperaments including the depressive temperament [53]. Indeed, affective temperaments, if dysregulated, could develop into abnormal affective pathologies that could emerge within mood disorders and affective disorders [74]. While the complexity of the relation between affective temperaments and mood episodes still prevails, research proved that there is an underlying role of a depressive temperament within unipolar major depression [74]. To add, researchers have shown that depression is a risk factor of PSU; when individuals are overwhelmed with negative emotions as a result of their depression, they have a tendency to rely on their smartphones as an escape strategy for the purpose of avoiding these negative emotions [18, 75, 76]. Nevertheless, PSU, as an escape strategy, has negative consequences since it does not give the outcome desired [77].

On the other hand, the findings of this study showed that higher self-esteem was significantly associated with a lower smartphone addiction rate. These findings support previous studies correlating low self-esteem with smartphone addiction [78] proving that self-esteem is a predictor of developing PSU [24, 56, 57, 79]. Davis elaborated a cognitive-behavioral model showing that PSU is at the core of developing the maladaptive cognition of the self, including low self-esteem [80]. Indeed, individuals who suffer from low self-esteem believe that they are held in higher esteem by other individuals during online interactions. Therefore, they rely on this modality to seek approval and recognition. Moreover, Greenberg et al. [81] developed the terror management theory of self-esteem: when individuals face a threatening situation that intimidates their self-esteem integrity, they engage in compensatory behaviors, such as PSU or other behavioral problems. Through the smartphone vessel, they can reach a virtual world to satisfy their self-esteem. Nonetheless, this PSU could lead to a vicious cycle. Another caveat highlighted by Yea and Kim where individuals with low self-esteem were inclined towards virtual interactions instead of in-person interactions in order to seek reassurance [82]. Consequently, this smartphone dependence could lead to a PSU as a way to fulfill these needs [24, 83, 84].

### Mediation analysis

The results of the mediation analysis, taking self-esteem as a mediating variable, showed that self-esteem fully mediated the association between depressive temperament and smartphone addiction. To understand the mediating effect of self-esteem between the depressive temperament and PSU, self-esteem should be considered as a risk factor of PSU [24, 56, 57]. A previous study associated depressive temperament with self-esteem, suggesting low self-esteem as a sub-factor to the depressive temperament [55]. However, individuals with depressive temperament have been defined by many researchers as people who adopt a self-denial mechanism to seek others’ approval and recognition and find harmony in social conformity [85]. Once deprived from these social roles, they become vulnerable to developing clinical depression. These behaviors prevailing social norms can be defined as social desirability [86]. To draw the link between the depressive temperament and self-esteem, social desirability is considered to be a strategy of self-regulation, allowing the subject to preserve a fragile self-esteem [87]. Furthermore, the Social Desirability Scale predicts a fragile self-esteem and a dependency to seek approval from others [88]. Hence, social desirability found in depressive temperaments is considered a self-regulating strategy allowing the subject to preserve a fragile self-esteem by responding to assumed social expectations [89]. In addition, subjects that self-associated as the object of favorable evaluation by others, had a better self-esteem. In fact, self-esteem cannot be conceived of apart from the reference to a social group. This is why many researchers consider it to be, above all, a reflection of the popularity feeling and the need of others’ approval: the level of self-esteem is highly correlated with the subjective experiences based on others’ approvals or rejections [79] depending on the level of social conformity, a key trait in the depressive temperament. Hence, individuals with low self-esteem seek approval and recognition from other individuals they interact with in the virtual world through PSU [80]. To add, Ozturk et al. indicated that depressive temperament and addiction to the Internet are correlated [53]. Therefore, people with depressive temperament with high social desirability tend to preserve a fragile self-esteem by developing PSU to satisfy their need of belonging and obtaining recognition.

This study’s results also showed that self-esteem fully mediated hyperthymic temperament and smartphone addiction. To understand the mediating effect of self-esteem between the hyperthymic temperament and PSU, Ozturk et al. [53] showed that hyperthymic temperament and addiction to the Internet are correlated. So far, one previous study associated hyperthymic temperament with self-esteem, suggesting high self-esteem was placed as a sub-factor of the hyperthymic temperament [55], while another study showed a direct correlation between hyperthymic temperament and self-esteem amongst patients suffering from bipolar disorder [90]. Hyperthymic temperament is related to type A behavior pattern [91] involving a strong need to compete against other people because accomplishments are evaluated as more valuable than circumstances and rules [92]. In addition, hyperthymic temperament has been described as partially having reckless and selfish traits [93]. Karam et al. found that people with hyperthymic temperament would purposefully engage in conflicts with the people around them rather than agree with the opinion of the majority [94]. Although one of the traits of this temperament is “having self-confidence”, other traits such as “liking to be the boss”, “getting into heated arguments”, and “the right and privilege to do as I please” reflect what Karam et al. called ‘the dark side of the hyperthymic temperament’ [94]. Indeed, from one side it protects the individual from suicidal behavior, but from the other side it corroborates the development of certain mental disorders. To draw the link between the dangerous effect of ‘high self-esteem’ as a trait of the hyperthymic temperament and PSU, studies have shown that a high level of self-esteem protects the person from developing PSU. However, subjects suffering from PSU tend to seek instant answers to preserve their self-esteem [95]. It could be noted that in order not to lose their high but fragile self-esteem, subjects with hyperthymic temperament develop PSU. Therefore, self-esteem has a mediating effect between hyperthymic temperament and PSU.

## Clinical implications

This study could help experts, in a psychotherapeutic or psychological support setting, gain a better insight of the psychological functioning of the Lebanese adult population. When dealing with individuals with a high SAS score and / or problematic self-esteem level, therapists could refer to the findings in this study. As such, practitioners could help the subject improve identifying and prevent the negative implications of affective temperaments, problematic self-esteem and PSU while having a deeper knowledge of the mediating interrelations between these factors. When faced with a clinical demand to reduce PSU, therapeutic interventions on these specific temperaments could, therefore, focus on alleviating problematic self-esteem and thereby reduce the level of PSU.

## Limitations and strengths

There are several limitations to this study that are worth mentioning to preserve the scientific integrity of this study and assist future research regarding this topic. The data's cross-sectional nature limits the ability to pull causal conclusions between our three variables. The use of a self-administered questionnaire poses a risk for information bias since the answers can sometimes be random due to question misinterpretations. There is also a risk of selection bias, given the snowball sampling technique in this study. A residual confounding bias is possible since not all factors associated with problematic smartphone use were taken into consideration in this study. Nevertheless, in spite of these limitations, this research reported interesting results encouraging additional exploration of smartphone addiction, which is a significant issue in this population. These results can be extrapolated to the whole population according to the recruitment method followed. To end, in our review of literature and to our knowledge, we found no previous studies investigating the mediating effect of self-esteem between affective temperaments and PSU. This posed a challenge in comparing this study’s results to those of previous studies, but on the other hand, it enhanced the importance and the uniqueness of this study.

## Conclusion

To better understand the high smartphone addiction rate among young adults in Lebanon, this study uses a valid SAS to highlight the mediating self-esteem in the association between depressive and hyperthymic temperaments with problematic smartphone use. Future studies in other countries can yield comparative results that could also be useful in complementing this study. A cause and effect study could also be implemented to better understand the relationship dynamic between these three variables. Finally, studying different types of therapeutic interventions on affective temperaments may be beneficial in reducing the rate of PSU in Lebanese young adults. In fact, what matters most is not only depicting the associative factors behind PSU among adults in Lebanon, but also developing therapeutic plans and evaluating their efficiencies in the subject’s growth towards a healthier mental health hygiene and optimal quality of life.

## Supplementary Information

**Additional file 1**. Full details of the mediation analysis taking self-esteem as a mediator between each temperament and smartphone addiction.

## Data Availability

The datasets generated and/or analysed during the current study are not publicly available as per their institutions’ policies but are available from the corresponding author on reasonable request.

## Abbreviations

SAS-SV: Smartphone addiction scale—short version
TEMPS-A: Affective temperament scale
RSES: Rosenberg self-esteem scale
PSU: Problematic social media use
DSM-5: Diagnostic and statistical manual for mental disorders
