# Supplementary material for: Problematic smartphone use and affective temperaments among Lebanese young adults: scale validation and mediating role of self-esteem
Source: BMC Psychol. 2021 Sep 8;9:136. doi: 10.1186/s40359-021-00638-y (PMC8424409; doi:10.1186/s40359-021-00638-y)
Supplement: Supplementary file 1 — Additional file 1. Full details of the mediation analysis taking self-esteem as a mediator between each temperament and smartphone addiction. [file 40359_2021_638_MOESM1_ESM.docx]

| **Supplementary Table 1. Mediation analysis: Self-esteem as a mediating variable and each temperament as a dependent variable.** | | | | | | | | | | |
| --- | --- | --- | --- | --- | --- | --- | --- | --- | --- | --- |
|  | **Effect of the temperament on self-esteem** | | | **Effect of temperament and self-esteem on smartphone addiction** | | | **Effect of temperament on smartphone addiction** | | | **Mediating effect of self-esteem** |
|  | **Beta**  **[95% BCa]** | **T** | **p** | **Beta**  **[95% BCa]** | **t** | **P** | **Beta**  **[95% BCa]** | **t** | **p** |  |
| Depressive temperament | -0.44  [-0.57- -0.32] | -7.06 | **<0.001** | 0.21  [-0.05-0.47] | 1.59 | 0.112 | 0.34  [0.09-0.58] | 2.66 | **0.008** | 60.15% |
| Self-esteem |  |  |  | -0.28  [-0.47- -0.10] | -3.05 | **0.002** |  |  |  |  |
|  | | | | | | | | | | |
| Cyclothymic temperament | -0.18  [-0.28- -0.09] | -3.67 | **<0.001** | 0.14  [-0.06-0.34] | 1.42 | 0.157 | 0.20  [-0.002-0.40] | 1.94 | 0.052 | - |
| Self-esteem |  |  |  | -0.28  [-0.47- -0.10] | -3.05 | **0.002** |  |  |  |  |
|  |  | | |  | | |  | | |  |
| Hyperthymic temperament | 0.54  [0.45-0.63] | 11.89 | **<0.001** | -0.07  [-0.28-0.13] | -0.69 | 0.491 | -0.23  [-0.41- -0.05] | -2.46 | **0.014** | 215.16% |
| Self-esteem |  |  |  | -0.28  [-0.47- -0.10] | -3.05 | **0.002** |  |  |  |  |
|  | | | | | | | | | | |
| Irritable temperament | -0.002  [-0.11-0.10] | -0.03 | 0.978 | 0.12  [-0.09-0.33] | 1.10 | 0.270 | 0.12  [-0.09-0.33] | 1.10 | 0.272 | - |
| Self-esteem |  |  |  | -0.28  [-0.47- -0.10] | -3.05 | **0.002** |  |  |  |  |
|  | | | | | | | | | | |
| Anxious temperament | 0.004  [-0.08-0.09] | 0.10 | 0.923 | 0.08  [-0.09-0.24] | 0.93 | 0.351 | 0.08  [-0.09-0.24] | 0.91 | 0.362 | - |
| Self-esteem |  |  |  | -0.28  [-0.47- -0.10] | -3.05 | **0.002** |  |  |  |  |
